# Tauroursodeoxycholate—Bile Acid with Chaperoning Activity: Molecular and Cellular Effects and Therapeutic Perspectives

**DOI:** 10.3390/cells8121471

**Published:** 2019-11-20

**Authors:** Magdalena Kusaczuk

**Affiliations:** Department of Pharmaceutical Biochemistry, Medical University of Białystok, Mickiewicza 2A, 15-222 Białystok, Poland; mkusaczuk@wp.pl; Tel.: +48-85-748-56-90

**Keywords:** TUDCA, bile acids, ER stress, apoptosis, cytoprotection, inflammation

## Abstract

Tauroursodeoxycholic acid (TUDCA) is a naturally occurring hydrophilic bile acid that has been used for centuries in Chinese medicine. Chemically, TUDCA is a taurine conjugate of ursodeoxycholic acid (UDCA), which in contemporary pharmacology is approved by Food and Drug Administration (FDA) for treatment of primary biliary cholangitis. Interestingly, numerous recent studies demonstrate that mechanisms of TUDCA functioning extend beyond hepatobiliary disorders. Thus, TUDCA has been demonstrated to display potential therapeutic benefits in various models of many diseases such as diabetes, obesity, and neurodegenerative diseases, mostly due to its cytoprotective effect. The mechanisms underlying this cytoprotective activity have been mainly attributed to alleviation of endoplasmic reticulum (ER) stress and stabilization of the unfolded protein response (UPR), which contributed to naming TUDCA as a chemical chaperone. Apart from that, TUDCA has also been found to reduce oxidative stress, suppress apoptosis, and decrease inflammation in many in-vitro and in-vivo models of various diseases. The latest research suggests that TUDCA can also play a role as an epigenetic modulator and act as therapeutic agent in certain types of cancer. Nevertheless, despite the massive amount of evidence demonstrating positive effects of TUDCA in pre-clinical studies, there are certain limitations restraining its wide use in patients. Here, molecular and cellular modes of action of TUDCA are described and therapeutic opportunities and limitations of this bile acid are discussed.

## 1. Introduction

**Figure 1:**
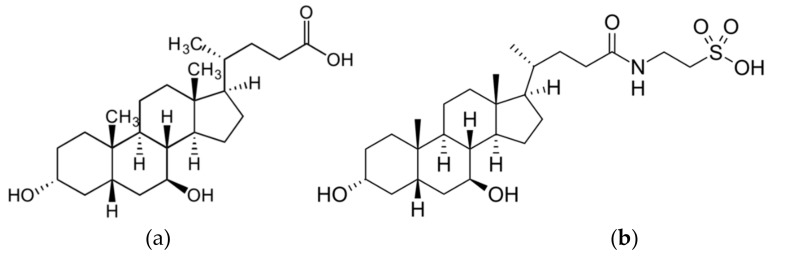
Chemical structures of secondary bile acids. (**a**) ursodeoxycholic acid; (**b**) tauroursodeoxycholic acid.

For centuries, traditional Chinese medicine has valued animal bile for its use in pharmacological and clinical applications. Bile acids are natural products and fundamental components of bile [1]. Tauroursodeoxycholic acid (TUDCA) is a taurine conjugate of ursodeoxycholic acid (UDCA) (Figure 1) [2]. Based on its choleretic activity and capability of protecting hepatocytes, UDCA has been approved by FDA (US Food and Drug Administration) for treatment of primary biliary cholangitis (previously primary biliary cihrrosis) [3,4]. The advantage of bile acids in therapeutic use is that they might be administrated via oral, subcutaneous, and intravenous routes of application. Furthermore, in most cases they present rather low toxicity to the organism and are able to cross the blood-brain barrier [4]. These favorable characteristics of TUDCA are the main reason why it has been studied as a potential therapeutic agent in a broad spectrum of diseases. 

**Figure 2:**
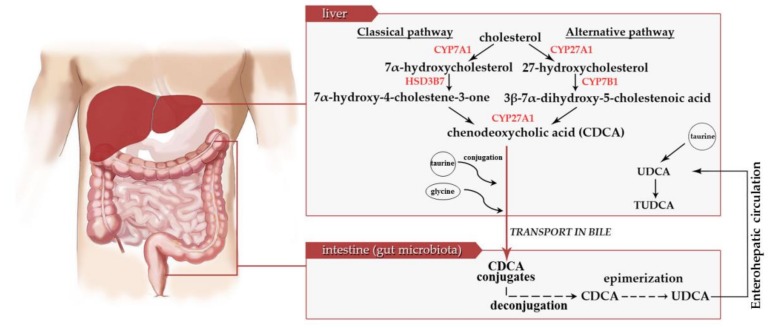
Biosynthesis of tauroursodeoxycholic acid. Detailed description in the text. CYP7A1—microsomal cholesterol 7a-hydroxylase; CYP27A1—27-hydroxylase; HSD3B7—3β-hydroxy-Δ5-C27-steroid dehydroxylase; CYP7B1—oxysterol 7α-hydroxylase; UDCA—ursodeoxycholic acid; TUDCA—tauroursodeoxycholic acid.

UDCA is a secondary bile acid that can be produced exclusively by intestinal microbiota [2]. Human liver is able to synthesize from cholesterol only primary bile acids such as chenodeoxycholic acid (CDCA) and cholic acid, which are further conjugated with glycine or taurine and secreted in bile in a form of taurocholic acid, glycocholic acid, glycochenodeoxycholic acid, and taurochenodeoxycholic acid. Further processing of primary bile acids is performed by gut bacteria and finally results in the formation of secondary bile acids such as UDCA [3,5]. The main pathway of TUDCA biosynthesis includes the conversion of cholesterol to 7α-hydroxycholesterol by microsomal cholesterol 7α-hydroxylase (CYP7A1). Next, 7α-hydroxycholesterol is converted to 7α-hydroxy-4-cholestene-3-one by 3β-hydroxy-Δ5-C27-steroid dehydroxylase (3β-HSD, HSD3B7). This is followed by the series of reactions including steroid side chain cleavage catalyzed by the mitochondrial sterol 27-hydroxylase (CYP27A1) to form chenodeoxycholic acid [4]. Alternatively, cholesterol might be transformed to 27-hydroxycholesterol by CYP27A1, then hydroxylated to 3β-7α-dihydroxy-5-cholestenoic acid by oxysterol 7α- hydroxylase (CYP7B1), and finally converted by CYP27A1 to CDCA [4]. CDCA and its conjugates are then secreted with bile into the large intestine where gut microbiota transforms it into UDCA. Finally, UDCA returns to the liver with enterohepatic circulation and is there conjugated with taurine to form TUDCA [5]. The main steps of TUDCA biosynthesis are presented in Figure 2. 

The series of enzymatic reactions gives secondary bile acids certain specific features not only regarding their hydrophilicity and lipophilicity, but also determining their capability of binding and activating certain receptors [6]. Thus, signaling pathways of bile acids may be initiated basically via two types of receptors, membrane as well as nuclear ones [6]. Given this, TUDCA/UDCA has now been recognized as a ligand of membrane receptors such as Takeda G-protein receptor 5 (TGR5) and sphingosine-1-phosphate receptor 2 (S1PR2) [7]. It has also been suggested that TUDCA/UDCA is transported into the cell via Na^+^/taurocholate co-transporter peptide (NTCP) and is then able to bind α5β1 integrin, transforming it into its active conformation by transferring β1 subunit [8,9,10]. Moreover, after internalization, TUDCA/UDCA can also activate nuclear receptors such as farnesoid X receptor (FXR) and glucocorticoid receptor (GR) [7]. 

Recently, the activity of TUDCA has been demonstrated to extend beyond hepatobiliary disorders. Progressing development of modern societies results in increased risk of so-called civilization diseases which include i.e., diabetes, obesity, cardiovascular diseases, or cancer [11]. Most of these disorders are in fact molecularly connected with endoplasmic reticulum (ER) stress and disrupted protein folding machinery. Thus, a group of pharmacological agents possessing potentially beneficial effects against ER stress are known as ‘chemical chaperones’ [12,13,14,15,16,17]. TUDCA is one such compound that is known for its chaperoning activity [12,13,14,15]. Until now, various studies have demonstrated good efficiency of TUDCA in alleviating or resolving ER stress, playing a role as a chemical chaperone; however, the exact chemical interactions involved in such activity are still debatable [18,19,20,21,22,23].

Moreover, TUDCA has been demonstrated to exert its cytoprotective activity not only by mitigating ER stress but also by preventing apoptosis [24,25,26,27,28,29,30]. Thus, it has been demonstrated that TUDCA/UDCA-mediated cytoprotection might be dependent on prevention of the translocation of pro-apoptotic Bax from cytosol to mitochondria [25,28]; inhibition of cytochrome c release and subsequent suppression of mitochondrial apoptosis [30]; and reduction of the expression of cyclin D1, suggesting involvement in cell cycle control [31,32]. Other mechanisms of action have also been proposed. Therefore, TUDCA/UDCA has been shown to interfere with E2F-1/Mdm-2/p53 apoptotic pathway [32,33,34,35] and to promote dissociation of heat shock protein 90 (HSP90) from mineralocorticoid receptor (MR) and GR, resulting in subsequent nuclear translocation of bile acid-receptor complex and reduction of apoptosis [36,37].

Finally, activation of pro-survival pathways seems to be an important mechanism supporting anti-apoptotic properties of TUDCA/UDCA [38,39]. Indeed, activation of anti-apoptotic nuclear transcription factor (NFκB) [40], stimulation of mitogen-activated protein kinase (MAPK)- and phosphatidylinositol 3-kinase (PI3K)-dependent pathways [29,41], or initiation of Akt signaling cascade [42], have already been demonstrated.

In light of the available knowledge, TUDCA arises as a potent therapeutic agent in a variety of human diseases. This review is focused on demonstrating molecular and cellular effects of TUDCA in hepatobiliary and ER stress-related disorders and tries to highlight its therapeutic potential and certain limitations restraining its use as an effective therapeutic agent in a broad spectrum of diseases.

## 2. TUDCA as Bile Acid in Hepatobiliary Disorders

Bile plays an essential physiological role in the human body. It is produced by hepatocytes to take part in fat digestion. Abnormalities in bile secretion can result in digestion problems and serious hepatobiliary diseases [43]. It is believed that exogenous administration of bile components can be a reasonable therapeutic approach improving outcomes of cholestatic liver diseases [43]. One of the bile components with beneficial effects in alleviating liver damage in a setting of cholestasis is UDCA [44,45]. UDCA was first synthesized in 1950 and successfully introduced into clinical treatment of liver diseases [43,46]. Benefits of using UDCA in a wide range of hepatobiliary disorders such as primary sclerosing cholangitis (PSC), pediatric cholestatic disorders, primary biliary cholangitis (PBC), and drug-induced cholestasis, have been evaluated in a series of clinical studies [47,48,49,50,51]. Although in patients with PBC, UDCA has been demonstrated to significantly improve liver function, its use in PSC remains more controversial. Not only is this therapy barely effective in reducing liver enzymes, but also there are no positive effects on long-term survival and side effects of high doses of UDCA are serious enough not to suggest its routine use in PSC [52].

It is assumed that UDCA mode of action is connected with the promotion of membrane insertion of transporters for biliary lipids into hepatocyte bile canaliculus [45]. Indeed, administration of UDCA resulted in improved biochemical parameters in patients suffering from nonalcoholic steatohepatitis [53,54] and resulted in more rapid reduction of bilirubin concentration and transaminases levels in patients with drug-induced liver injury [55]. Furthermore, UDCA presented good efficacy in improvement of the outcome of primary biliary cholangitis mostly due to increased hydrophilicity of the biliary bile acid pool [56,57]. 

It has been known that cytotoxicity of bile acids decreases with their increasing hydrophilicity. Thus, conjugation with taurine makes UDCA more polar, which accounts for higher therapeutic effectiveness of TUDCA [58,59,60]. Compared to UDCA, TUDCA is better absorbed by intestine and liver because of being fully ionized and water soluble at various pH values [60]. Recent multicenter randomized clinical trials have shown that TUDCA presents the same level of safety and tolerability as UDCA for the treatment of primary biliary cholangitis and may be even better to relieve symptoms of the disease, suggesting higher effectiveness of taurine conjugate in treatment of PBC [61]. Colell et al. demonstrated that TUDCA can not only be effective in improving the outcome of cholestatic liver diseases, but also display hepatoprotective potential during long-term exposure to ethanol in rats [62]. TUDCA supplementation prevented tumor necrosis factor α (TNFα)-induced peroxide formation and hepatic cell death, suggesting modulation of mitochondrial membrane fluidity and subsequent normalization of mitochondrial glutathione levels as a novel mechanism of TUDCA-mediated hepatoprotection in ethanol-fed rats [62].

Despite many promising systemic effects, the mechanism of anti-cholestatic and choleretic activity of TUDCA/UDCA at the molecular level is still incompletely understood. Cytoprotection of injured cholangiocytes against the toxic effects of bile acids seems to be the predominant mechanism of action in early-stage PSC and PBC, while post-transcriptional stimulation of disrupted hepatocellular secretion seems to be relevant in more advanced cholestasis [63,64,65]. The first reports suggesting that UDCA can play the role of an intracellular signaling molecule appeared in early 1990s [66,67,68,69]. UDCA was then implied to be an activator of protein kinase C (cPKCα) [70,71,72], an agonist of Ca^2+^ [66,67,68,69], an activator of mitogen-activated protein kinases (MAPK; Erk1/2 and p38^MAPK^) [73,74], and a stimulator of α5β1 integrins in hepatocytes and cholangiocytes [75,76]. On the other hand, TUDCA was demonstrated to augment the secretory capacity of hepatocytes and cholangiocytes by stimulation of vesicular exocytosis through Ca^2+^-/cPKCα-dependent vesicle fusion process and insertion of key transport proteins into their target membrane [66,70]. Later research revealed that TUDCA exerts its choleretic activity via a dual p38^MAPK^/integrin-dependent mechanism of apical carrier insertion [73,76] and stimulation of Cl^−^ secretion in cholangiocytes through the activation of membrane Ca^2+^-activated Cl^−^ channel (TMEM16A) [77]. In contrast, TUDCA exerts its anti-cholestatic properties mainly by enhancing secretory capacity of hepatocytes [78]. This effect was found to be caused by stimulation of exocytosis and insertion of the apical conjugate export pump—Mrp2 (multidrug resistance-associated protein 2) into canalicular membranes of rat cholestatic hepatocytes [78]. The mechanisms by which TUDCA exerted its hepatoprotective effect involved Ca^2+^ mobilization and activation of cPKCα-dependent pathways [78,79,80]. In line with these reports, Dombrowski et al., demonstrated that in an experimental model of cholestasis, TUDCA markedly increased canalicular density of another pivotal apical transporter, the bile salt export pump (ABCB11) [81]. Altogether, these results indicate that the stimulation of apical membrane insertion of key transporter proteins such as Mrp2 and ABCB11 may contribute to the anti-cholestatic activity of TUDCA in cholestatic conditions.

**Figure 3:**
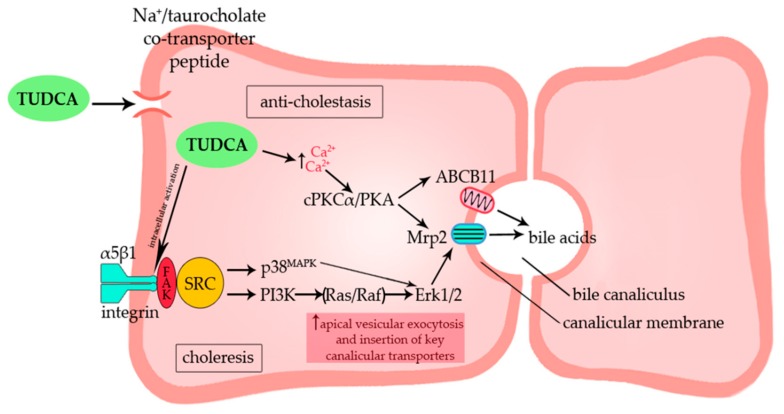
Schematic presentation of cellular effects and molecular signaling pathways initiated by TUDCA in cholestatic hepatocytes. Hepatoprotective effects are attributable to the TUDCA-mediated activation of two main signaling pathways: Ca^2+^-dependent signaling pathway and α5β1 integrin-dependent signaling pathway. Stimulation with TUDCA increases the insertion of key canalicular bile acid transporters resulting in anti-cholestetic and choleretic effect. Mrp2—multidrug resistance-associated protein 2; ABCB11—bile salt export pump; cPKCα—protein kinase C; PKA—protein kinase A; FAK—focal adhesion kinase; SRC—steroid receptor co-activator; p38^MAPK^—mitogen-activated protein kinase; PI3K–phosphoinositide-3-kinase; ERK1/2—extracellular signal-regulated kinase.

It has been known that TUDCA evokes cellular effects after entering the hepatocyte, without affecting extracellular membrane/receptor interactions [9]. Thus, liver cells expressing NTCP would show higher sensitivity to TUDCA than any other cell type [9,82]. Gohlke et al. have found that in perfused rat liver and human NTCP-transfected HepG2 hepatoma cells, TUDCA unlike other bile acids caused rapid transfer of the β1 unit of mainly cytosolic α5β1 integrins into its active conformation, leading to phosphorylation and activation of Erk1/2, epidermal growth factor receptor and further downstream signaling cascade [9]. Therefore, β1 integrin was suggested to be the main cellular sensor of TUDCA [8,9]. The overview of the molecular effects of TUDCA/UDCA in cholestasis is summarized in Figure 3.

In conclusion, despite a considerable amount of research suggesting that the application of TUDCA/UDCA may be an effective strategy in managing the outcomes of hepatobiliary disorders, clinical trials have not fully confirmed their efficiency in suffering patients. Therefore, unraveling the exact signaling pathways underlying TUDCA effectiveness may be an essential step in designing future therapeutic strategies aimed at improving the response of cholangiopathies to bile acid-based pharmacotherapy in human individuals. 

## 3. TUDCA as Chemical Chaperone in ER Stress-Related Diseases

Maintaining equilibrium of the ER is an essential element of proper cell functioning. In resting “conditions’’, ER receptors are kept inactive by binding to the ER molecular chaperone GRP78 (78-kDa glucose-regulated protein). However, homeostasis of the ER might easily be interrupted by various stimuli resulting in a state known as ER stress [12,83]. In ER stress, normal protein processing is hindered, which results in an accumulation of unfolded or misfolded proteins in the ER lumen [83,84]. In order to cope with this impaired protein processing, a cascade of signal transduction called unfolded protein response (UPR) is initiated [83]. UPR is controlled by three transmembrane sensors which are: PKR-like ER kinase (PERK), inositol-requiring enzyme 1α (IRE1α), and activating transcription factor 6 (ATF6) [12,83,84]. When the ER homeostasis is disrupted, GRP78 detaches from these three receptors, which initiates further downstream signaling cascades leading to the alleviation of misfolded proteins overloaded mainly by decreasing the protein synthesis to reduce burden of misfolded proteins, enhancing biosynthesis of molecular chaperones, and activating a proteasomal degradation process called the ER-associated degradation (ERAD) pathway [83,84]. The primary aim of the UPR is therefore an adaptive pathway, however, when the stress is severe and “insuperable’’, UPR may activate a pro-apoptotic branch of response through the up-regulation of transcription factor CCAAT/enhancer-binding protein homologous protein (CHOP) [83]. Recently, the malfunction of the ER and subsequent accumulation of unfolded/misfolded protein aggregates are thought to be molecular hallmarks of many diseases collectively known as conformational diseases [82,83]. Thus, in humans, defective protein folding is believed to be one of the key molecular hallmarks of disorders, such as diabetes, obesity, neurodegeneration, or cancer [83,84].

In light of this knowledge, it seems reasonable to assume that drugs defeating ER stress may be promising candidates for the treatment of multiple conformational diseases [16,17,85]. On the basis of functional analogy to molecular chaperones, this group of molecules was called “chemical chaperones’’. Chemical chaperones are usually defined as low-molecular-weight compounds restoring the proper destination of incorrectly localized and/or aggregated proteins mostly by stabilizing their structure and facilitating their folding [14,85,86]. As such, glycerol, trimethylamine N-oxide, dimethyl sulfoxide and 4-phenylbutyrate have been identified as chemical chaperones [12,13,87]. Lately, numerous studies have confirmed that TUDCA is also able to exert chaperoning-like activity in various cell types [19,20,85,86]. 

Until now, a precise mode of action of chemical chaperones has not been determined. Most likely, these molecules may exert their effects through structural stabilization of improperly folded proteins, stimulation of molecular chaperones to more effective protein trafficking, reduction of protein aggregation, and protection against nonspecific protein–protein interactions [14,88,89,90]. Moreover, they were suggested to decrease the energy barrier between conformational states during protein maturation to promote proper protein folding and to bind the surface-exposed hydrophobic elements of misfolded/unfolded proteins to prevent aggregate formation and degradation in ERAD pathway [87,91,92]. 

Although the exact mechanism of chaperoning activity of TUDCA is still unclear, it has been shown to prevent UPR malfunction and ameliorate ER stress in various cell types [21,22,93,94,95]. TUDCA exerts these effects partially by assisting in the transfer of mutant proteins and partially by improving protein folding capacity through the activation of ATF6 [93]. It has also been reported to effectively mitigate stress-induced aggregation of bovine serum albumin (BSA) in HepG2 cells by enhancing trypsin-mediated digestion of this protein [85] and to act through the activation of the pro-survival PERK/eIF2α/ATF4 pathway [96,97]. 

Overall, it seems that the main goal of using chemical chaperones is to mimic functioning of the molecular ones and support restoration of ER homeostasis. Since ER stress-dependent signaling intersects with molecular pathways underlying many other cellular pathological processes such as oxidative stress and inflammation, chemical chaperones may act as cytoprotectants and promising therapeutic agents in the therapy of many diseases. Thus, regarding cytoprotective, anti-apoptotic, and anti-inflammatory effects of TUDCA, this bile acid was investigated by many researchers as a potential candidate for the treatment of many disorders.

### 3.1. TUDCA in Diabetes and Obesity

In modern societies, diabetes mellitus and intercurrent illnesses are some of the major causes of morbidity and mortality worldwide [98]. Type 1 diabetes is characterized by loss of insulin-producing pancreatic β islets mainly as a result of autoimmunological aggression [12,84]. Type 2 diabetes is typically associated with normal insulin production, but lower insulin sensitivity of cells, which in consequence leads to elevated glucose levels in the blood [12,84]. An increasing number of reports from experimental and clinical studies also indicate a higher prevalence of cardiac damage [98], obesity [99], vision impairment [23,100], and nephropathy [101,102] in diabetic patients. According to a growing number of data, ER stress is a key contributor to the pathology of diabetes and is associated with pancreatic beta cell loss and insulin resistance [103,104]. Moreover, the occurrence of ER stress has been demonstrated in pathology of obesity. In humans, augmented ER stress existed not only in adipose tissue of obese insulin-resistant patients, but also in obese non-diabetic individuals, suggesting chemical chaperones to be promising candidates for novel therapeutics for both diabetes and obesity [105,106]. Indeed, increasing evidence postulates that TUDCA evokes beneficial effects in the outcome of diabetes. Recent studies suggest that TUDCA is effective in e.g., increasing insulin sensitivity [99], potentiating insulin clearance [107], and increasing pancreatic beta-cell mass [103] in obese humans and murine models of obesity and diabetes. TUDCA has been demonstrated to prevent palmitate-induced apoptosis in rat pancreatic β-cell line INS-1 and to evoke beneficial effects on acinar cells in the experimental model of acute pancreatitis mostly by reducing ER stress [108,109]. TUDCA also decreased tunicamycin-induced expression of protein tyrosine phosphatase 1B (PTP1B) in cultured myotubes [110] and reduced arterial stiffness in aortic and perivascular adipose tissue of type 2 diabetic mice [111]. All these effects were demonstrated to occur as a result of the alleviation of ER stress as demonstrated by decreased expressions of GRP78, ATF4, p-eIF2α, XBP-1 and CHOP, and reduced activation of caspases-12 and -3 [109,110,111]. 

In obese humans, TUDCA improved insulin sensitivity of muscle and liver, but not adipose tissue [99]. This effect was accompanied by increased insulin-stimulated phosphorylation of IRS^Tyr^ and Akt^Ser473^ in muscle, however, without causing such alterations in adipose tissue. These results suggest that although ER stress-mitigating activity of TUDCA has been widely demonstrated in in-vitro and animal model studies of diabetes and obesity, research conducted on humans may not fully confirm these findings. Thus, further analyses are required to confront the results of experimental models with the molecular effects of TUDCA administration in human individuals. 

The hallmarks of type 1 diabetes are decreased insulin production, enhanced glucose production, and hyperglycemia, which occur due to loss and dysfunction of pancreatic β-cells [112]. Despite the undeniable role of ER dysfunction in type 2 diabetes, its role in the pathogenesis of autoimmune type 1 diabetes remains elusive. Nevertheless, defects in the expression of the UPR mediators such as ATF6 and XBP1 have been identified in β cells from both mouse models of type 1 diabetes as well as type 1 diabetic patients [112]. Administration of TUDCA led to the restoration of expression of the UPR mediators and resulted in reduced β cell apoptosis, increased β cell mass, higher β cell number per islet, and maintained insulin secretion together with marked reduction in blood glucose [103,112]. These data suggest that adequate maintenance of the UPR might be a pivotal factor for the preservation of β cells, and chemical restoration of imbalanced ER homeostasis can be used for therapeutic or preventive interventions in type 1 diabetes. 

Although the ER stress-alleviating effect of TUDCA has been already well established, molecular signaling pathways engaged in the process of restoration of cellular homeostasis in diabetes remain elusive. It is believed that bile acids interact with several signaling pathways including membrane-bound bile acid receptors e.g. TGR5, and nuclear receptors e.g. FXR, which are known to regulate the metabolism of glucose and lipids and to control bile acid transportation and turnover [101,113,114]. As such, TUDCA-mediated activation of FXR resulted in enhanced glucose-stimulated insulin secretion (GSIS) in isolated islets of knockout mice, which was accompanied by a concomitant increase in Ca^2+^ influx, inhibition of ATP-dependent K^+^ (K_ATP_) channels, and modulation of electrical activity of β-cells [114]. In contrast, in high glucose-incubated isolated islets of C57Bl/6 mice, TUDCA was demonstrated to increase GSIS, without changing glucose metabolism or altering Ca^2+^ signals and K_ATP_ channel activity. This suggested that TUDCA enhances GSIS in pancreatic β-cells likely by activating the TGR5 receptor and initiating the cAMP/PKA pathway [113]. Additionally, TUDCA was demonstrated to be a major helper in counteracting obesity-induced hyperinsulinemia in the liver of high-fat-diet-fed C57Bl/6 mice due to improved insulin clearance [107]. This effect was a result of TUDCA-mediated increase in the expression of insulin-degrading enzyme most probably via bile acid receptor S1PR2 activation and initiation of its downstream signaling cascade involving insulin receptor/PI3K/Akt pathway [107]. Also, TUDCA was shown to suppress TNF-α-induced lipolysis in 3T3-L1 adipocyte cell line via reduction of ER stress and initiation of the IRE-JNK-perilipin-A signaling pathway [115]. Altogether, reduced ER stress and subsequent alleviation of lipolysis and hyperinsulinemia may count for the preventive effect of TUDCA against obesity and type 2 diabetes [107,115]. 

Lately, in addition to the well-known modulatory effect of TUDCA on ER stress and UPR signaling, a novel mechanism of functioning of this bile acid attributed to the regulation of autophagy has been proposed [102,116]. Guo at al., demonstrated that TUDCA improved glycolipid metabolism disorder in the liver of obese C57BL/6J mice via alleviation of ER stress and restoration of defective hepatic autophagy [116]. These findings were partially confirmed by Fang et al., who demonstrated that under diabetic conditions autophagy in the kidney podocytes was inhibited, and when treated with TUDCA, the glomeruli isolated from mice showed improved autophagy and reduced ER stress [102]. Nevertheless, both studies suggested that engagement of TUDCA in the regulation of autophagy may not be its primary mode of action, but may rather occur as a secondary effect of ER stress-modulatory activity. Thus, since there is a clear crosstalk between autophagy and ER stress, more emphasis should be put into examination of the role of TUDCA on modification of the autophagy process when using this bile acid to treat ER stress-related diseases.

Interestingly, the latest studies suggest a novel UPR-independent mechanism of TUDCA beneficial activity in type 1 diabetes, consisting of the regulation of micronutrients metabolism [117,118]. These studies imply an important role of Mg^2+^ and Ca^2+^ [118], as well as Cu^2+^ and Zn^2+^ [117] in glycemic control in type 1 diabetic mice models. It has been shown that levels of these microelements were altered significantly during the progress of diabetes, and long-term administration of TUDCA restored Mg^2+^, Ca^2+^, Cu^2+^, and Zn^2+^ levels to normal values [117,118]. Unfortunately, mechanisms underlying micronutrient regulatory activity of TUDCA remain unknown, as well as the exact significance of this modulatory effect on the outcome of diabetic individuals. It may be speculated that this might be connected with antioxidant and ER stress-alleviating potential of TUDCA, since Cu^2+^ and Zn^2+^ can act as structural and catalytic components of some metalloproteins such as SOD, whereas Ca^2+^ in particular is responsible for maintaining proper organelle functioning and initiation of stress responses [119,120].

A set of recent research have shown that TUDCA may also be beneficial in controlling the outcome of certain obesity/diabetes-related disorders. It has been found to present a favorable effect in morbidities such as cardiac failure [98,104,121] and vision impairment [23,100]. In a mice model of obesity-induced myocardial dysfunction, treatment with TUDCA resulted in increased expression and activity of sarco(endo)plasmic reticulum Ca^2+^-ATPase (SERCA) and alleviation of augmented levels of p-IRS^Ser307^, cJun, p-cJun, p-JNK, and ER stress markers [98,104,121]. This was followed by improvement in cardiac hypertrophy, diastolic diameter, fractional shortening, cardiomyocyte contractile, and diminished cardiac fibrosis [98,104,121]. 

Beneficial effects of TUDCA have also been suggested in studies of diabetic cataract [23] and diabetic retinopathy [100]. TUDCA was demonstrated to markedly retard the occurrence of cataract due to reduced ER stress and diminished oxidative stress [23]. In a rat model of diabetic retinopathy, TUDCA protected retinal cells against high glucose-dependent apoptosis in an ER stress-independent manner. This anti-apoptotic effect was in part mediated by inhibition of the release of apoptosis inducing factor (AIF) from the mitochondria, and in part by reduction of ROS production and prevention of oxidative stress [100]. These results may indicate that the cytoprotective effect of TUDCA may extend beyond ER stress-mitigating mechanisms, becoming a potentially beneficial molecule in resolving oxidative stress-dependent pathologies. 

Altogether, these data demonstrate the pleiotropic effects of TUDCA on correcting deficits associated with type 1/type 2 diabetes and obesity. Nevertheless, mitigation of ER stress seems to be a predominant mechanism of action of TUDCA, confirming its chemical chaperoning activity. These data suggest that this bile acid might be a promising pharmaceutical candidate for treatment of metabolic disorders. However, further analyses are still needed to fully unravel molecular, cellular and systemic effects of TUDCA in order to use it successfully as a therapeutic agent in diabetes-suffering patients.

### 3.2. TUDCA in Neurodegenerative Diseases

In humans, defective protein folding is thought to be connected with many neurodegenerative diseases [12,122]. In this respect, TUDCA not only has been shown to inhibit apoptosis induced by several stimuli in neuronal cells in vitro, but also to play a cytoprotective role in animal models of neurological disorders, such as Alzheimer’s disease (AD) [6,35,123,124,125], Parkinson’s disease (PD) [3,6,126,127,128,129,130], Huntington’s disease (HD) [24,131], amyloid latheral sclerosis (ALS) [132,133,134], and Prion diseases [135]. Furthermore, pretreatment with TUDCA significantly reduced glutamate-induced apoptosis of rat cortical neurons [38] and improved synaptic plasticity as well as cognitive and motor impairment in the hippocampus of microcystin–leucine–arginine-treated rats [136,137]. 

However, the overall TUDCA mode of action in neuropathologies seems to be considerably different to that observed in the case of other chemical chaperones such as PBA [12]. The molecular basis of the neuroprotective potential of TUDCA is rather focused on its anti-apoptotic properties more than ER stress-alleviating activity [24,37,126,131,138,139]. First, reports concerning TUDCA functioning in neurodegenerative diseases come from the studies of Alzheimer’s disease. At the molecular level, AD is characterized by the formation of the neurofibrillary tangles (NFTs) and deposition of amyloid-β (Aβ) plaques, which is followed by neuronal loss due to enhanced apoptosis [6,124,125,126]. Early studies demonstrated that TUDCA was efficient in reducing neuronal cell death induced by unconjugated bilirubin and Aβ plaques [140]. These results have been confirmed by further findings demonstrating anti-apoptotic properties of TUDCA to be exerted by the reduction of nuclear fragmentation; suppression of caspase 2 and 6 activation; and modulation of p53, Bcl-2 and Bax activity, suggesting inhibition of the E2F-1/p53/Bax pathway as a key element of TUDCA-mediated cytoprotection [35,41,140,141]. Furthermore, TUDCA has also been suggested to affect neuronal death via an alternative apoptotic pathway connected with the activation of nuclear steroid receptors, such as the glucocorticoid receptor and the mineralocorticoid receptor (MR) present in the brain [37]. Pretreatment with TUDCA significantly influenced Aβ peptide-dependent changes in nuclear steroid receptors, causing dissociation of MR from its cytosolic chaperone HSP90 and its further translocation into the nucleus [37]. Lately, novel research demonstrated that TUDCA attenuates amyloid precursor protein (APP) processing, reduces Aβ deposition and prevents cognitive impairment in APP/PS1 mice [124,125]. It has been shown that TUDCA decreases the accumulation of cerebral Aβ deposits causing amelioration of memory deficits and modulated γ-secretase activity resulting in decreased amyloidogenic APP processing [124,125]. As a consequence, a marked reduction in Aβ1-40 and Aβ1-42 levels was noticed in both the frontal cortex and hippocampus of TUDCA-fed APP/PS1 mice, suggesting that TUDCA might interfere with Aβ formation possibly via regulation of lipid-metabolism mediators connected with APP processing [124,125]. These results suggest that TUDCA supplementation might be considered as a potential therapeutic strategy for the prevention and treatment of Alzheimer’s disease.

The second most common neurodegenerative disorder worldwide is Parkinson’s disease [127]. PD is characterized by the loss of dopaminergic neurons in the *substantia nigra* of the brain, which results in severe motor symptoms such as dyskinesia, tremor, and speech impairment [3,127,128,129,130]. Dopamine cell death in PD may result from either genetic or environmental factors. Despite the different etiology, increased oxidative stress is assumed to take part in the neurodegenerative process in both familial and sporadic PD [142,143,144,145]. Since TUDCA is known to counteract oxidative stress and mitochondrial damage, it seems promising to use it as potential neuroprotective agent in Parkinson’s disease [140,143,144]. It has been demonstrated that in sodium nitroprusside (SNP)-treated SH-SY5Y cells, UDCA decreased ROS generation, diminished the production of reactive nitrogen species, inhibited loss of mitochondrial membrane potential (MMP), and facilitated maintenance of the intracellular glutathione (GSH) levels, resulting in reduced cell death [144]. Furthermore, TUDCA prevented 1-methyl-4-phenyl-1,2,3,6-tetrahydropyridine (MPTP)-induced neurodegeneration in a mouse model of PD [27]. It has been discovered that TUDCA protects against MPTP-induced dopaminergic degeneration by preventing JNK phosphorylation and reducing ROS production, thus suggesting that the TUDCA-mediated survival pathway involves Akt signaling, followed by downstream phosphorylation of Bad and activation of NF-κB [27]. Accordingly, TUDCA was demonstrated to prevent 1-methyl-4-phenylpyridinium (MPP+)-induced damage in primary mouse cortical neurons [127]. Pre-incubation with TUDCA protected against MPP+-induced dysfunction of mitochondria and neuronal cell death, mainly via increased accumulation of full length PINK1 and mitochondrial localization of parkin. TUDCA also exerted a modulatory effect on parkin phosphorylation in Ser65 position. Since PINK1-mediated phosphorylation of parkin is an important event in neuronal mitophagy, TUDCA was then suggested to exert its neuroprotective effects through the modification of PINK1/parkin-mediated mitophagy [127,146,147]. Other reports suggested that TUDCA prevented oxidative stress mainly via enhanced expression of Nrf2, DJ-1, and antioxidant enzymes heme oxygenase-1 (HO-1) and glutathione peroxidase (GPx) [128]. The latest studies show that the MPTP model of PD TUDCA restored ATP levels, prevented mitochondrial dysfunction, alleviated neuroinflammation, and significantly improved motor symptoms [130]. Currently, a novel approach towards the utilization of bile acids in PD is being considered. Since, to date, no reliable biomarkers of PD exist, Graham et al. investigated alterations in bile acid profiles as potential biomarkers used in early diagnosis of PD [129,148]. In this respect, prodromal mouse model of PD was used to measure serum concentrations of 18 bile acids. Three bile acids were identified to have markedly different concentrations, including ω-murichoclic acid (MCAo), UDCA, and TUDCA. Interestingly, TUDCA and UDCA showed the most pronounced down-regulation (17-fold and 14-fold decrease respectively), suggesting potential usefulness of TUDCA/UDCA in predicting susceptibility to PD [129]. Although still preliminary, these results warrant further investigations concerning the potential role of TUDCA/UDCA as reliable indicator of PD. Overall, these data point out that pharmacological benefits of TUDCA in neuroprotection may contribute to the validation of this bile acid in clinical application for treatment of PD.

TUDCA may also be effective in diminishing the outcomes of other neurodegenerative diseases such as HD [24,131,139], ALS [132,133,134], prion diseases [135], and retinal disfunctions [149,150]. In mouse models of HD, TUDCA has been shown to suppress cytochrome c release, decrease caspases activation, inhibit DNA fragmentation, and reduce the number and volume of striatal lesions, resulting in enhanced neuronal survival and improved sensorimotor and locomotor deficits [24,131,139]. However, despite several promising reports demonstrating favorable effects of TUDCA in HD models, it has never been clinically tested for having beneficial effects in patients. Thus, further intensive investigations are still necessary to provide evidence for TUDCA activity in HD suffering individuals. In contrast, clinical testing of TUDCA/UDCA was performed in ALS. Clinical trials indicated potential effectiveness, safety, penetration into the cerebrospinal fluid, and overall good tolerance of these bile acids in patients [132,133,134]. Nevertheless, although safe and well tolerated, TUDCA has not yet been approved for treatment of ALS and further more extensive examinations need to be provided to confirm its potential efficacy in this disease. 

In prion diseases, the accumulation of misfolded mutated prion protein (PrPSc) causes astrogliosis, loss of neurons, and spongiform brain degeneration which results in ataxia, dementia, and finally death of the affected individual. One of the therapeutic approaches for these diseases is targeted at blocking or interfering with the conversion of normal prion protein into PrPSc [135]. TUDCA and UDCA were shown to significantly reduce such conversion, thus diminishing neuronal loss, prolonging neuronal survival and reducing astrocytosis in prion-infected male C57BL/6 mice [135]. These results suggest that TUDCA/UDCA may present a therapeutic potential in management of prion diseases via engagement of both neuroprotective and prion protein conversion-dependent mechanisms.

In terms of visual disorders, bear bile has been used as a therapeutic agent in Chinese medicine for ages [150]. Given this, TUDCA has been investigated to evaluate its efficacy against several ophthalmological diseases [151]. In retinitis pigmentosa (RP), TUDCA was demonstrated to preserve synapses between photoreceptors and horizontal or bipolar cells, and reduce photoreceptor loss across the retina of rats [152]. Also, when administrated subcutaneously, TUDCA caused a marked reduction in apoptosis, thickened outer the nuclear layers of retina, increased the number of photoreceptor cells, facilitated the development of photoreceptor outer segments, and overall, substantially slowed retinal degeneration [143,153,154,155]. In Leber congenital amaurosis (LCA), mutations in either lecithin-retinol acyltransferase (LRAT) or retinoid isomerase (RPE65) lead to S-opsin aggregation and subsequent ER stress, which in turn results in rapid degeneration of cone cells [156]. In experimental model of LCA, TUDCA treatment retarded ventral and central cone degeneration by enhancing the degradation of cone membrane-associated proteins via the ERAD pathway and alleviation of CHOP activation, suggesting that TUDCA protects cones by restoring ER homeostasis [156]. The neuroprotective effect of TUDCA against cell death has also been observed in retinal ganglion cells after visual and chemical stimulation [157,158] and in experimental retinal detachment [150]. After retinal detachment, TUDCA prevented photoreceptor death by diminishing oxidative stress, reducing the activity of pro-apoptotic caspases-2, -3 and -9, without affecting ER stress and caspase-11 nor -8 levels [150]. Moreover, TUDCA enhanced phagocytosis of the photoreceptor outer segment and protected against H_2_O_2_-induced phagocytic dysfunction in both cultured ARPE-19 cells and primary human retinal pigment epithelium (hRPE) cells [159]. Interestingly, TUDCA seemed to evoke a cytoprotective effect independently of ER stress, as presented by the unchanged GRP78 expression in tunicamycin-treated ARPE-19 cells [159]. 

Overall, the molecular basis of the cytoprotective effect of TUDCA is connected with its anti-apoptotic activity [35,41,140,144] and ability to reduce ROS production [128], rather than resolving ER stress in neuronal cells. Although, in a multitude of research, TUDCA is basically claimed to act as chemical chaperone, studies do not seem to confirm that resolving ER stress is a predominant molecular mode of action of this bile acid in neurodegenerative diseases. Thus, further investigations are still necessary to get comprehensive knowledge about TUDCA mode of action. Studying the neuroprotective potential of TUDCA is even more encouraging since bile acids were demonstrated to possess the capability of penetrating the blood-brain barrier [133,135] and to have a high level of safety when administrated to patients in clinics [135]. Given this, TUDCA arises as a novel promising therapeutic agent in treatment of neurological disorders.

### 3.3. TUDCA in Inflammation

It is already well established that inflammatory responses are activated in many types of diseases including hepatic ischemia reperfusion (HIR) [160], asthma [161], neurological disorders [162,163], or Sjögren’s syndrome (SS) [164]. Moreover, a crosstalk between inflammation and ER stress exists, in a way that UPR pathways intersect with a multitude of stress and inflammatory signaling networks including the JNK-AP-1-, NFκB-, and IκB kinase (IKK)-dependent pathways [12]. This may suggest, that in addition to the ER stress-mitigating effect, TUDCA can also alleviate inflammatory responses via UPR-dependent signaling. Indeed, such dependencies have been demonstrated in several reports concerning different types of inflammation-related disease models. It has been demonstrated that in mesenchymal stem cells (MSCs), *P*-cresol-induced ER stress resulted in apoptotic and inflammatory responses [165]. Upon *P*-cresol exposure, pro-inflammatory mediators such as p-NF-κB, p-JNK, and p-p38 were significantly overexpressed. Treatment with TUDCA alleviated ER stress and decreased inflammation. Interestingly, these cytoprotective effects of TUDCA were abolished when prion protein (PrPc) was knocked down, suggesting a PrPc-dependent mode of action [165]. Altogether, these results suggested that the anti-apoptotic and anti-inflammatory effects of TUDCA are indeed preceded by mitigation of ER stress. 

Xu et al., demonstrated that in a mouse model of hepatic ischemia reperfusion (HIR), injury TUDCA attenuated HIR injury as shown by both the improvement of liver functioning in vivo and decrease in hepatocyte apoptosis in vitro [160]. TUDCA diminished the expression and secretion of pro-inflammatory cytokines IL-1β, IL-6 and TNF-α by suppressing ER stress in Kupffer cells via the IRE1α/TRAF2/NF-κB pathway [160]. In a mice model of steatohepatitis, C57BL/6 mice treated with TUDCA showed limited hepatocyte lipoapoptosis and steatohepatitis, while in-vitro TUDCA decreased palmitate-induced ER stress significantly reduced inflammation score and down-regulated the expression of pro-inflammatory cytokines Ccl2 and TNFα [166]. Likewise, in a non-alcoholic fatty liver disease (NAFLD) model, TUDCA alleviated gut inflammatory responses via down-regulation of pro-inflammatory cytokines e.g., IL-1β, Ccl2, Ccl4, and Icam1 and improved intestinal barrier function by increasing levels of tight junction molecules and the solid chemical barrier [167]. Also, TUDCA supplementation caused significant attenuation of expression of pro-inflammatory mediators such as cyclooxygenase (COX), phospholipase A2 (PLA2), and prostaglandin E2 (PGE2) in tunicamycin-treated rats and this effect was most likely dependent on the alleviation of ER stress [168].

Modulation of ER stress has also been considered to be engaged in anti-inflammatory properties of TUDCA in allergic airway disease [161,169] and Sjögren’s syndrome [164]. TUDCA decreased ER stress and attenuated pulmonary levels of the pro-inflammatory cytokines IL-6 and IL-33 and chemokines CCL20/macrophage inflammatory protein 3 α (MIP3α), C-C motif chemokine ligand 11 (CCL11/eotaxin-1), and CXCL1/keratinocyte-derived chemokine (KC) in mice [161]. Also, treatment with TUDCA exerted a beneficial outcome in reversing aberrant mucin 1 (MUC1) accumulation in salivary glands of SS-suffering individuals [164]. In experimental conditions, TUDCA reduced TNF-α and IFN-γ-induced up-regulation of pro-inflammatory markers such as TNF-α, IL-1β, and IL-6, and alleviated NF-κB activation [164]. Interestingly, contradictory modes of action of TUDCA have been further suggested. Preferential direct binding to ATF6α receptor and alleviation of ER stress have been proposed as primary effects of TUDCA in a murine model of allergic airway disease [161]. In contrast, ER stress-relieving activity of TUDCA has been suggested to be a secondary effect of reduced expression of pro-inflammatory mediators, rather than its primary mode of action in Sjögren’s syndrome [164]. This suggests that TUDCA-mediated interplay between ER stress and inflammatory responses might be more complex and further analyses are still necessary to comprehensively solve this issue.

Although a great deal of research focusing on the ER stress-dependent modulatory effect of TUDCA on inflammatory responses exists, several studies investigating the anti-inflammatory potential of this bile acid regardless of its ER stress-modifying activity have also been performed [162,163,170,171]. However, only one report clearly presented that TUDCA abolished salt-induced renal injury and inflammation in endothelin B receptor (ETB)-deficient rats independently on ER stress response, implying different mechanisms of its anti-inflammatory effects [171]. Indeed, TUDCA treatment did not decrease measures of ER stress as presented by the unchanged expressions of GRP78, ATF-4, ATF-6, sXBP-1, CHOP, and caspase-12, however, it markedly reduced salt-induced TNF-α excretion and CD4+ T lymphocyte accumulation in the renal cortex [171]. Interestingly, although other studies also clearly presented anti-inflammatory properties of TUDCA, its influence on the modulation of ER stress has not been evaluated [162,163,170]. Thus, TUDCA not only was shown to inhibit expressions of pro-inflammatory mediators such as nitric oxide (NO), inducible nitric oxide synthase (iNOS), TNF-α, IL-1β, and cyclooxygenase-2 (COX-2), but also to induce the expression of anti-inflammatory mediator, arginase 1 [170]. Moreover, TUDCA was demonstrated to inhibit activation of NF-κB in both astrocytes and microglia, causing diminution of iNOS and a subsequent decrease in the release of nitrite by these cells [162]. In this respect, alternative modes of anti-inflammatory action of TUDCA independent of UPR signaling have been proposed. TUDCA has been suggested to reduce leukocyte extravasation to the inflammation site in the CNS due to reduced expression of chemoattractants (e.g., MCP-1) and vascular adhesion proteins (e.g., VCAM-1) [162], and to act through regulation of the transforming growth factor β (TGF-β) expression [163]. Nevertheless, in spite of the indisputable anti-inflammatory potential of TUDCA, engagement of ER stress-dependent signaling cannot be excluded. Thus, molecular pathways involved in TUDCA-mediated anti-inflammatory responses cannot be clearly stated. Although certain suggestions concerning the UPR-independent mode of action exist, lack of evaluation of the ER stress-modulatory effect in latter studies makes it impossible to exclude such involvement. 

Altogether, these findings suggest that TUDCA may be a promising candidate as a therapeutic agent in inflammatory diseases, however, further studies are necessary to fully recognize the therapeutic potential of this bile acid to confirm its mode of action and use it as successful treatment/co-treatment in inflammation.

### 3.4. TUDCA in Cancer

Malignant diseases are characterized by the development of abnormal cells that are capable of dividing uncontrollably and infiltrate and destroy normal body tissue. In the context of quickly proliferating cells, an enhanced demand for protein synthesis exists, thus ER is a key organelle guarding proper cellular functioning in cancer [84,172]. In terms of oncotherapy, the role of the UPR signaling in malignant cells may be controversial, since the pro-survival branch of the UPR aims at rescuing transformed cells. Nevertheless, if activated, the pro-apoptotic pathway results in death and elimination of neoplastic cells. As such, therapeutic approaches concerning ER stress in cancer would rather focus on enhancing ER stress than on restoring ER homeostasis. Given this, some contemporary chemotherapeutic strategies in oncology concentrate on targeting ER stress as a potential goal of pharmacological agents [173,174]. Thus, it seems counterintuitive to use chemical chaperones such as TUDCA in cancer research, since their cytoprotective potential would rather benefit in maintaining neoplastic cells instead of eliminating them. Paradoxically, several reports from in-vitro and in-vivo studies inform that TUDCA/UDCA might have some favorable effects in therapy of certain types of cancer [175,176,177,178,179,180,181]. Early studies of the influence of TUDCA on growth of human cholangiocarcinoma Mz-ChA-1 cell line demonstrated that TUDCA suppressed the growth of the Mz-ChA-1 cells due to increased calcium signaling and downstream activation of PKC-α- and MAPK p42/44-dependent pathways [175]. 

Also, anti-proliferative and pro-apoptotic activity of UDCA has been shown in human melanoma M14 cell lines [179]. In M14 cells, UDCA activated the intrinsic pathway of apoptosis and caused G2/M cell cycle arrest. Simultaneously, UDCA showed little toxicity to normal human cell lines: LO2 hepatocytes and HaCaT keratinocytes [179]. Nevertheless, these results although promising, should be treated with caution since the highly pro-apoptotic potential of UDCA was limited to M14 cells and was not that satisfactory in A375, another melanoma cell line [179]. Therefore, further studies concerning different cell lines are needed to confirm these preliminary data.

Studies conducted on hepatocellular carcinoma and colitis-associated colon cancer (CACC) have shown that anti-inflammatory activity of TUDCA may underlie its anti-cancer potential [176,181]. In carcinogen-induced liver dysfunction and hepatocellular carcinoma, TUDCA presented chemopreventive properties by preventing ER stress and alleviating hepatic inflammation [176]. Whereas, in a mice model of CACC, TUDCA-mediated diminution of cancer development was dependent on the reduction of phosphorylated IκB kinase and extensive epithelial apoptosis in the colon of treated animals [181]. Additionally, in TNF-α-stimulated HCT116 cells, TUDCA markedly decreased the expression of IL-8 and IL-1α and inhibited TNF-α-induced phosphorylation/degradation of IκBα together with suppression of NF-κB DNA-binding activity [181]. These studies suggest that TUDCA is able to ameliorate tumorigenesis mostly via alleviation of NF-κB-mediated inflammatory response, thus paving the way for chemopreventive use of this bile acid in oncogenesis of certain carcinomas. 

On the other hand, Park et al. suggested anti-metastatic potential of TUDCA [177]. In MDA-MB-231 breast cancer cells cultured in normoxic and hypoxic conditions, treatment with TUDCA caused significant down-regulation of MMP-7 and MMP-13 mRNAs in both experimental conditions resulting in marked decrease in invasion but not migration of MDA-MB-231 cells [177]. This effect was indicated to be dependent on PERK signaling, suggesting TUDCA as a tentative ER stress-targeting candidate for anti-metastatic agents in breast cancer. 

Taking into consideration the cytoprotective activity of TUDCA/UDCA, it has been used as a co-therapeutic agent helping to deal with the outcomes of standard chemotherapeutic approaches [178]. UDCA when administrated to Sprague-Dawley rats treated with 5-fluorouracil (5-FU) protected animals against chemotherapy-induced intestinal mucositis, which is a common side effect of chemotherapy [178]. As such, UDCA administration alleviated 5-FU-induced body weight loss, reduced inflammatory cytokine (TNF-α and IL-6) levels, and attenuated intestinal villus damage, suggesting beneficial effects of chemotherapy-associated mucositis [178]. 

Altogether, TUDCA/UDCA have been demonstrated to possess chemopreventive, anti-metastatic, and pro-apoptotic potential in cancer. However, although the results of preliminary studies suggest these bile acids to be beneficial in controlling the outcomes of certain malignant diseases, the knowledge concerning this issue remains elusive. Surprisingly, the influence of TUDCA/UDCA on ER stress and UPR signaling has only been marginally studied in the abovementioned research, which do not give the full insight into the molecular mode of action of these bile acids in transformed cells. Thus, using chemical chaperones in cancer studies remains controversial and a whole bunch of comprehensive analyses would have to be done in the future to consider this approach as a potential avenue in modern oncopharmacology.

## 4. Therapeutic Perspectives and Limitations

TUDCA/UDCA has been used in Chinese medicine for centuries, and hundreds of studies have already underscored its beneficial effects in the treatment of many pathological conditions. Nevertheless, TUDCA is constantly a very intriguing pharmacological agent and a growing number of pre-clinical and clinical research is still performed, unraveling novel aspects of TUDCA functioning [94,182,183,184,185,186,187]. Although through the years this bile acid has been demonstrated to be a very efficient hepatoprotectant and ER stress alleviator, many innovative contemporary reports inform about its novel potential applications and molecular modes of action. It has been discovered, that these new properties of TUDCA occur in addition to/or independently of ER stress-modulating potential. In this respect, in mice fed with a high-fat-diet, TUDCA was found to increase the expression of genes connected with β-oxidation (such as peroxisomal acyl-CoA oxidase 1 and long-chain acyl-CoA dehydrogenase) in the liver and suppress genes involved in fatty acid uptake (such as fatty acid translocase, fatty acid transport protein 4, and fatty acid receptor 3) in the intestine [167]. These findings, together with others reporting weight loss in obese mice [107], may suggest to further investigate TUDCA as potential candidate for weight-reducing agent.

Moreover, two recent reports have suggested beneficial effects of TUDCA in therapy of osteoarthritis [187,188]. Here, apart from rescuing chondrocytes from ER stress and ER stress-induced apoptosis, TUDCA has been shown to augment cyclin D1 expression and cell proliferation; increase levels of chondrogenic markers such as SOX9, COL2, ACAN, and chondroitin sulfate; attenuate intracellular cholesterol; increase membrane fluidity; and enhance the expression of focal adhesion proteins such as vinculin, integrin α5, and integrin β1. Together, these findings suggest that TUDCA could be used as an alternative treatment for the restoration of cartilage damage in osteoarthritis [187,188].

Intriguingly, the latest research suggests a completely novel aspect of TUDCA functioning consisting of the modification of epigenetic mechanisms in the cell [184,189,190]. In addition to ER stress-alleviating activity, in serum-starved nuclear donor cells, TUDCA markedly attenuated the expression levels of DNA methyltransferase 1 (DNMT1) and histone deacetylase 1 (HDAC1), resulting in a significant increase in the global level of acetylated histone 3 (H3K9ac) [184]. Epigenetic modulatory activity of TUDCA has also been suggested in an experimental model of prenatal ethanol exposure [189,190]. Here, adult offspring of affected animals suffered from increased ER and oxidative stress, insulin resistance, and glucose intolerance followed by augmented gluconeogenesis and reduced expression of muscle glucose transporter-4 (GLUT4). Additionally, expressions of HDAC1, 3, 4, 5, 7, and 9 were demonstrated to be significantly higher in alcohol-exposed animals. All these effect were shown to be reversed by TUDCA treatment [189,190]. Interestingly, TUDCA-dependent inhibition of class I and II HDAC enzymes restored the expression GLUT4, resulting in improvement of insulin sensitivity and glucose tolerance [189]. Moreover, TUDCA has been found to modify the expression of sirtuins, which are NAD-dependent deacetylases responsible for removing acetyl groups from many histone and non-histone proteins [185,190]. Thus, TUDCA decreased ethanol-induced overexpression of sirtuin-2 [190] and restored hypoxia- and hemorrhagic shock-induced down-regulation of sirtuin-1 [185]. It is commonly known that modulation of the epigenetic environment of the cell may interfere with a plethora of distinct cellular processes such as apoptosis, cell cycle, differentiation, or senescence, and also may affect the regulation of many metabolic pathways [191,192]. In this respect, it might be speculated that TUDCA-dependent epigenetic reprogramming may be one of the reasons for its pleiotropic effects in various cell types. However, the exact role of these modifications is not yet clear, which warrants further investigation of TUDCA-mediated changes of the epigenome.

With all that being said, not every piece of information concerning TUDCA/UDCA activity is so optimistic in pointing to these bile acids as a universal cure for a plenitude of diseases. Although in the vast majority of research TUDCA is presented as a very efficient and promising agent in pharmacological management of many disorders, there is one report saying that the utilization of UDCA beyond primary biliary cholangitis is unjustified [193]. The unsuspected side effects of UDCA treatment seem to include e.g., cholangitis, hepatitis, ascites, pruritus, vanishing bile duct syndrome, liver cell failure, severe watery diarrhea, immune-suppression, and mutagenic effects. On the molecular level, UDCA was implied to inhibit DNA repair, phagocytosis, and NOS induction, as well as suppress activation of co-enzyme A, cyclic AMP, and p53 [193]. Also, pro-carcinogenic potential of UDCA has been suggested [194]. Regarding this information, one should keep in mind that similar results have not been presented for TUDCA, however, close structural proximity of both bile acids raises some doubts and suggests treating the results of pre-clinical studies with sufficient caution. 

Additionally, when considering TUDCA as a potential therapy for non-hepatic disorders, one needs to be mindful of certain limitations. It has been known that TUDCA enters the cell through the Na^+^/taurocholate co-transporting peptide that is mainly expressed in hepatocytes. Given this, reduced ability of TUDCA uptake may exist in terms of other cell types, which may at least partially explain why TUDCA acts primarily in the liver [9]. Also, the route of administration into the organism seems to be an essential factor determining TUDCA effectiveness. In humans, basically only oral and intravenous administration of the drug can be considered. It has been suggested that the divergent efficacy of TUDCA treatment between mice and human subjects might be explained by insufficient plasma accumulation of TUDCA in patients taking TUDCA orally versus intraperitoneally injected mice (30 times more TUDCA relative to body weight was given to mice than to patients) [99]. Since TUDCA is effectively metabolized by the liver and only minimal splanchnic escape of bile acid conjugates into the systemic circulation, it is likely that a difference in route of administration may further limit the systemic availability of TUDCA in humans [195]. Additionally, there is still an insufficient amount of reports presenting the usage of TUDCA in patients not suffering from hepatobiliary disorders. When the use of TUDCA as a chemical chaperone in non-liver diseases is considered, its influence on a healthy hepatobiliary tract should also be addressed. All these speculations indicate that a thorough pharmacokinetic analysis of TUDCA needs to be performed to fully understand all limitations of its administration in human individuals. 

In light of the available knowledge, it may be stated that although a lot is already known about the biological effects of TUDCA/UDCA in a variety of different cell types and disease models, it is still a very attractive compound to investigate as a potential multifunctional pharmacological agent. This seems to be confirmed by constant progress in design and synthesis of novel and more active analogues as well as synthetic derivatives of these molecules [196,197,198]. Thus, despite certain limitations in utilizing TUDCA as a ready-to-use therapeutic approach in a wide variety of human morbidities, contemporary investigations bring high hopes for the development of efficient TUDCA-based therapeutics in the future.

## 5. Conclusions

Although TUDCA has been known to possess a multitude of activities including ER stress-modulatory, oxidative stress-modulatory, anti-apoptotic, anti-inflammatory, and supposedly epigenetic-modulatory activity in experimental conditions, the clinical relevance of these findings remains unknown and the exact molecular pathways activated after TUDCA treatment in humans are still to be identified. Despite a solid amount of data being already available for this bile acid, further extensive examinations are still performed, unraveling novel roles of TUDCA. This constant interest in unveiling TUDCA biology indicates that there are high expectations and hopes connected with therapeutic opportunities of this bile acid for future use as a natural alternative for many cytoprotective, chemopreventive, and anti-inflammatory drugs.
